# A knowledge-based structure-discriminating function that requires only main-chain atom coordinates

**DOI:** 10.1186/1472-6807-8-46

**Published:** 2008-10-29

**Authors:** Yoshihide Makino, Nobuya Itoh

**Affiliations:** 1 Department of Biotechnology, Faculty of Engineering, Toyama Prefectural University, 5180 Kurokawa, Imizu-shi, Toyama 939-0398, Japan

## Abstract

**Background:**

The use of knowledge-based potential function is a powerful method for protein structure evaluation. A variety of formulations that evaluate single or multiple structural features of proteins have been developed and studied. The performance of functions is often evaluated by discrimination ability using decoy structures of target proteins. A function that can evaluate coarse-grained structures is advantageous from many aspects, such as relatively easy generation and manipulation of model structures; however, the reduction of structural representation is often accompanied by degradation of the structure discrimination performance.

**Results:**

We developed a knowledge-based pseudo-energy calculating function for protein structure discrimination. The function (Discriminating Function using Main-chain Atom Coordinates, DFMAC) consists of six pseudo-energy calculation components that deal with different structural features. Only the main-chain atom coordinates of N, C_*α*_, and C atoms for the respective amino acid residues are required as input data for structure evaluation. The 231 target structures in 12 different types of decoy sets were separated into 154 and 77 targets, and function training and the subsequent performance test were performed using the respective target sets. Fifty-nine (76.6%) native and 68 (88.3%) near-native (< 2.0 Å C_*α *_RMSD) targets in the test set were successfully identified. The average C_*α *_RMSD of the test set resulted in 1.174 with the tuned parameters. The major part of the discrimination performance was supported by the orientation-dependent component.

**Conclusion:**

Despite the reduced representation of input structures, DFMAC showed considerable structure discrimination ability. The function can be applied to the identification of near-native structures in structure prediction experiments.

## Background

Protein structure evaluation is a key process in protein structure prediction, in association with comparative modeling, fold recognition, structure refinement, and *de novo *folding. Protein design technology also requires structure evaluation methods with sufficient capacity. Many different types of potential energy functions have been developed and examined. The formulation of the functions can be roughly grouped under physical-based and knowledge-based approaches [1,2,4]. Physical-based (or molecular mechanics) potential energy functions are mainly used for the simulation of protein folding and dynamics [2], and are also effective for protein design [3]. The knowledge-based approach to developing such an evaluation system is also effective and widely used, especially for protein structure prediction and protein design studies [1]. The classical approach is the extraction of "pseudo" mean potentials from the distribution of pairwise distances of known protein structures based on the Boltzmann law [5]. A number of potential constructions and their successful applications have been reported [5-16]. Recently, improved accuracy has been facilitated, accompanied with the accumulation of high-resolution protein structure information [17].

The assessment of pairwise distances is implemented in many knowledge-based functions. Several variations of atom types are utilized, such as C_*α *_and/or C_*β *_atoms [7], the center of mass of the side chain [16], and heavy atom representation for a variety of atom types [7]. The functions of other structural features, including hydrogen bonds [8], main-chain dihedral angles [14], and solvation potentials [6], were also reported. A number of functions have been formulated as a combination of the above functional components. The introduction of orientation-dependent components often improves the accuracy of the function. The hydrogen bond is a typical example, and the effectiveness of orientation-dependent potential was reported [8]. Buchete et al. introduced another type of orientation-dependent potential, using the pairwise interaction of local reference states for respective amino acids [9,10].

The structure discrimination capacity of the function is frequently estimated on the basis of the ability to correctly identify native or near-native structures from nonnative but plausible "decoy" structures. The "Decoys 'R' Us" database [18] is a collection of decoy sets, and is commonly used to evaluate functions. The database consists of 10 decoy sets, generated by different methods. Many other decoy sets, such as the "moulder" [15] or the "rosetta" [19], are also utilized to assess functions. It is commonly understood that the performance of structure evaluation functions tends to depend strongly on the intrinsic properties of decoy generation methods and/or other qualities of decoy sets [12]. Thus, many reports have assessed functions using multiple decoy sets and/or effective statistical techniques.

The compatibility of the structure-discriminating function for reduced structural representations provides many beneficial effects. For example, the generation and manipulation of model structures can be performed without more complexed structure construction; however, it is difficult to reduce the required structural information without losing the accuracy of the scoring function.

In this article, we report the development of a knowledge-based protein structure-discrimination function. The complexity of the required input structure data for evaluation was limited to the main-chain trace with only three atom coordinates (N, C_*α*_, and C) per respective amino acid residue. To overcome the possible loss of accuracy of decoy discrimination, orientation-dependent potential between two C_*α*_-pseudo-C_*β *_vectors was introduced. The parameter training and the subsequent performance test were carried out using the decoy sets from the Decoys 'R' Us database, in addition to the moulder and the rosetta decoy sets. High accuracy in native or near native structure recognition was observed in the test set. The level of discrimination ability was nearly comparable to other coarse-grained or all-atom-type functions. A detailed description of the development of the function and evaluation of the discrimination ability are provided.

## Results

### Function Design

**Figure 1:**
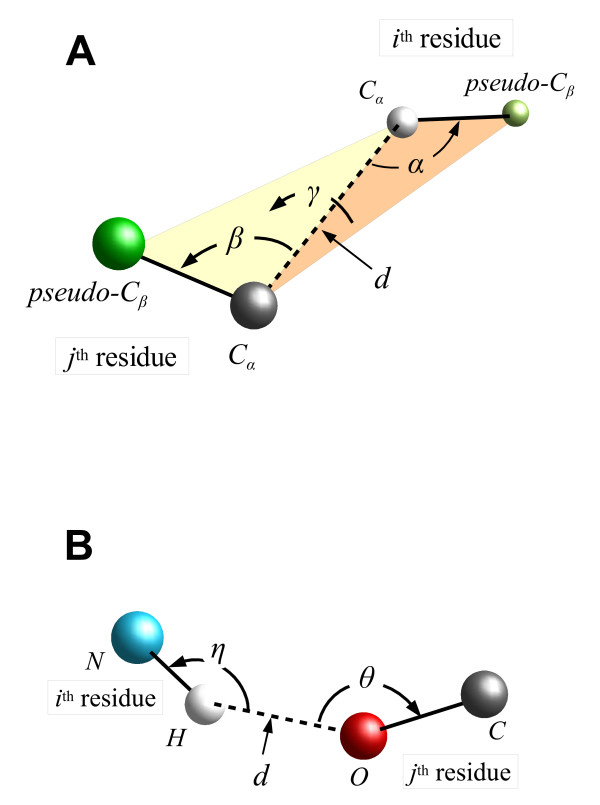
**Schematic representation of the pairwise residue parameters for pseudo-energy components**. (A) DABG component. Distance *d *(Å) is measured between two C_*α *_atoms. The *α *angle (degree) is formed with the C_*α*_-pseudo-C_*β *_vector of *i*^th ^residue and the C_*α*_-C_*α *_vector. The *β *angle (degree) is formed similarly for *j*^th ^residue. The *γ *is the dihedral angle (degree) formed with the four atom coordinates of the C_*α *_and the pseudo-C_*β *_for the respective *i*^th ^and *j*^th ^residues. (B) HBND component. Distance *d *(Å) is measured between pseudo-H atom of the *i*^th ^residue and pseudo-O atoms of the *j*^th ^residue. The *η *angle (degree) is formed with the pseudo-H-N vector of the *i*^th ^residue and the pseudo-H-pseudo-O vector. The *θ *angle (degree) is formed with the pseudo-O-C vector of the *j*^th ^residue and the pseudo-O-pseudo-H vector.

Before explaining the results of function development and structure evaluation, the overall design of the function is briefly described. The details of the function formulation can be found in Methods. The structure-discriminating function developed in this study consists of six pseudo-energy calculation components. Each of the components evaluates the distinctive structural feature of a target protein. The pseudo-energy is calculated based on the Boltzmann law [5], with knowledge-based procedures using a precompiled database from a non-redundant set of known structures. The six structural features focused on are as follows: the C_*α *_pairwise distance (the corresponding functional component is referred to as DIST), the relative orientation between two vectors of C_*α*_-pseudo-C_*β *_(DABG component, Figure 1A), hydrogen bonding between a main-chain amino hydrogen and a carbonyl oxygen (HBND component, Figure 1B), the main-chain dihedral angles of the combination between *ψ *at a residue and *φ *at the next residue (PPDA component), the main-chain *ω *dihedral angle (OMDA component), and the number of surrounding C_*α *_atoms around a C_*α *_atom (SURR component). Each atom coordinate is treated separately by twenty amino acid types. The overall function is formulated with the weighted linear combination of the above six pseudo-energy components. As the function was designed to require three main chain atom coordinates (amino nitrogen, C_*α*_, and carbonyl carbon) per residue as input data, we refer to the final form of the function as DFMAC (Discriminating Function using Main-chain Atom Coordinates).

### Function training with decoy sets

The parameters associated with each component and the weights of respective components are not inherently clarified. In order to search for and determine the parameter values, we used decoy sets.

The parameter values and weights were determined on the basis of the discrimination ability of the native structure from its decoys. The outline of the tuning procedure is as follows: (1) The probable set of values of the parameters was scanned and determined using arbitrarily collected decoy sets. (2) The parameters were further tuned using the training decoy set by the cross-validation procedure. (3) The weights of the respective function components were finally determined using the entire training set. The performance of the tuned function was evaluated using the test decoy set, which was distinctive from the training set. Details of the procedure are in Methods.

To determine the initial values of the parameters for the following tuning, we used 7 decoy sets of 4state_reduced, fisa, fisa_casp3, hg_structal, ig_structal, ig_structal_hires, and lmds from the Decoys 'R' Us database [18]. (Note: Although some targets used in the final performance test were included in these decoy sets, some of the parameter values of the respective components and weights were changed after the subsequent parameter tuning procedure (Table 1). Thus, bias in the final performance test is considered to be limited.) Parameters which decreased the average square values of C_*α *_RMSD of the best pseudo-energy structures for respective protein targets in the 7 decoy sets were successively selected.

**Table 1:** Parameters and their values for tuning the function.

scan	component	parameter	initial value	scanned values	selected value
1	DIST	*b*	12.0	9.0, 10.0, 11.0, 12.0, 13.0, 14.0, 15.0	12.0
		*c*	0.627	0.525, 0.550, 0.575, 0.603, 0.631, 0.661, 0.692	0.661
2	DIST	sequence separation limit	5	2, 3, 4, 5, 6, 7, 8	5
3	DABG	range of the bin averaging			
		distance	1	0, 1, 2	0
		*α *angle	0	0, 1	1
		*β *angle	0	0, 1	0
		*γ *angle	2	0, 1, 2	1
4	DABG	sequence separation limit	5	2, 3, 4, 5, 6, 7, 8	5
5	DIST, DABG	sequence separation limit of evaluation	3	1, 2, 3, 4, 5, 6, 7, 8, 9, 10	3
6	DIST	0 count penalty	8.0	0.0, 2.0, 4.0, 6.0, 8.0, 10.0, 12.0, 14.0, 16.0	8.0
7	DABG	0 count penalty	2.0	0.0, 1.0, 2.0, 3.0, 4.0, 5.0	2.0
8	SURR	radius range	15.0	9.0, 12.0, 15.0, 18.0	15.0
9	SURR	0 count penalty	0.0	0.0, 1.0, 2.0, 3.0, 4.0, 5.0, 6.0, 7.0	0.0
10	HBND	0 count penalty	2.0	0.0, 1.0, 2.0, 3.0, 4.0, 5.0, 6.0, 7.0	2.0
11	PPDA	0 count penalty	12.0	0.0, 2.0, 4.0, 6.0, 8.0, 10.0, 12.0, 14.0, 16.0	12.0
12	OMDA	0 count penalty	6.0	0.0, 2.0, 4.0, 6.0, 8.0, 10.0, 12.0, 14.0, 16.0	0.0

Short descriptions of scanned parameters of the components are shown in the order of scanning during the tuning process. The *b *and *c *of scan 1 are the constants for calculating *N*_exp_(*d*). Sequence separation limit of scan 2 and 4 are the lower limit of separation between *i*^th ^and *j*^th ^residues that was incorporated into the respective databases. The four parameters of scan 3 are the range for averaging among adjacent database bins. The sequence separation limit of evaluation of scan 5 is the lower limit of separation applied for evaluation, not for database construction. The 0 count penalties of scan 6, 7, 9, 10, 11 and 12 are the energy penalty value when no count was recorded in the bin of the compiled database. The radius range of scan 8 is the radius of the sphere for SURR component calculation. Details of the respective parameters are in Methods. The values determined by initial scanning before tuning, the list of scanned values during tuning, and the selected values with better C_*α *_RMSD are shown. Multiple parameters in a single scan indicate the scanning of all combinations among the listed values.

Using the probable parameters determined above as the initial parameter set, further tuning was then carried out. The 231 targets of the 10 decoy sets from the Decoys 'R' Us database, the moulder decoy set [15,20], and the all-atom decoy set from Rosetta@home , were separated into 77 and 154 targets and used for testing and training (or parameter tuning) the function, respectively. (Note: Each set consisted of targets from a variety of decoy sets.) Tuning was performed by 10-fold cross validation using the training set. Briefly, nine of ten parts of targets were used for parameter training, and then the function was validated with the remaining part of the targets. Performance with a distinct parameter set was evaluated by the C_*α *_RMSD average of the top structures from 10 evaluations with different training and validation combinations. After successive tuning of the parameters, the new parameter set was obtained (Table 1, see Methods). Finally, the weights of the respective function components were determined on a whole training decoy set with the new parameter set. Some of the parameters and weights were changed from the initial values during the above procedure.

A summary of the performance of the tuned function on the training set is shown in Table 2. Of 154 training targets, 115 (74.7%) native and 135 (87.7%) near-native (i.e. < 2.0 Å C_*α *_RMSD) structures were correctly identified as the best energy structures. The averages of the Z-score, correlation coefficient (C.C), and fraction enrichment (F.E.) were sufficiently positive. The performance on decoy structures, without native structures, is critical, because no native or near-native structure is available prior to structure prediction experiments. Thus, analyses were also carried out on decoys without native structures. Discrimination performance of decoy structures were also positive as indicated by the average values of logP_B1_, logP_B10_, and the correlation coefficient (C.C._decoy_) and fraction enrichment (F.E._decoy_) among decoy structures.

**Table 2:** Summary of performance of the DFMAC function on the training and test decoy sets.

target set	N_all_	N_n_	N_nn_	C_*α *_RMSD	Z-score	C.C.	F.E.(%)	R_B1_	logP_B1_	R_B10_	logP_B10_	C.C._decoy_	F.E._decoy_(%)
training set	154	115	135	0.764	2.552	0.539	38.9	171.3	-0.78	36.1	-1.38	0.499	27.6
test set	77	59	68	1.174	2.630	0.559	38.7	164.0	-0.75	15.8	-1.41	0.518	25.1

Summarized values are shown by the respective decoy sets. The numbers of total target proteins evaluated (N_all_), and correct identification of the native (N_n_) or near-native (C_*α *_RMSD < 2 Å) (N_nn_) structures are shown. The C_*α *_RMSD, Z-score, C.C., F.E., R_B1_, logP_B1_, R_B10_, logP_B10_, C.C._decoy_, F.E._decoy _are the average of the respective scores of the target proteins evaluated. The definition of each index is described in Methods.

### Decoy discrimination performance test

The performance of the tuned function cannot be evaluated on the training set itself, because versatility is not necessarily assured because of the possibility of over-learning; therefore, the structure discriminating ability of the tuned DFMAC was tested on the above test set, containing different targets from the training set. The results are summarized in Table 2, and the details are shown in Table 3. A large number of native structures of the respective protein targets were correctly identified as the best-energy (i.e. the lowest energy value) structures (Table 2). Correct identification of the native structures was 59 out of 77 targets (76.6% success), and the identification of near-native structures (C_*α *_RMSD < 2 Å) was 68 (88.3% success). The possible interpretations of failed identification of the remaining 9 targets are discussed below. The significantly positive average values of Z-score, C.C., and F.E. indicate considerable overall performance. The averages of the respective decoy discrimination scores (logP_B1_, logP_B10_, C.C._decoy_, and F.E._decoy_) were also significantly positive. Although the average C_*α *_RMSD of top-energy structures was 1.174, which was a little larger than the average on the training set, the percentage of correctly identified native or near-native structures was similar to the training set. Additionally, other indexes were also similar between the training and test sets. Thus, a certain degree of versatility was confirmed.

**Table 3:** Performance of the DFMAC function on the test decoy sets grouped by their generation methods.

protein	N	R_nat_	C_*α *_RMSD	Z-score	C.C.	F.E.(%)	R_B1_	logP_B1_	R_B10_	logP_B10_	C.C._decoy_	F.E._decoy_(%)

4state_reduced												

1ctf	631	1	0.000	4.485	0.817	68.3	61	-1.01	3	-2.32	0.815	66.7
2cro	675	1	0.000	3.166	0.822	53.7	7	-1.98	2	-2.53	0.820	53.7
4rxn	678	1	0.000	2.895	0.670	65.7	71	-0.98	3	-2.35	0.665	64.2
**Average**	**661.3**	**1.0**	**0.000**	**3.515**	**0.770**	**62.6**	**46.3**	**-1.33**	**2.7**	**-2.40**	**0.767**	**61.5**

fisa												

2cro	501	1	0.000	4.190	0.280	24.0	3	-2.22	2	-2.40	0.253	24.0
**Average**	**501.0**	**1.0**	**0.000**	**4.190**	**0.280**	**24.0**	**3.0**	**-2.22**	**2.0**	**-2.40**	**0.253**	**24.0**

fisa_casp3												

1bl0	972	8	5.522	2.174	0.302	20.6	15	-1.81	15	-1.81	0.296	19.6
l30	1401	1	1.882	3.835	0.128	18.6	455	-0.49	3	-2.67	0.111	17.9
**Average**	**1186.5**	**4.5**	**3.702**	**3.005**	**0.215**	**19.6**	**235.0**	**-1.15**	**9.0**	**-2.24**	**0.204**	**18.7**

hg_structal												

1bab-B	30	1	0.000	1.868	0.904	66.7	10	-0.46	2	-1.16	0.892	0.0
1ecd	30	1	0.000	1.364	0.896	100.0	2	-1.16	2	-1.16	0.898	50.0
1gdm	30	1	0.000	2.395	0.880	33.3	4	-0.86	2	-1.16	0.845	0.0
1hbh-B	30	1	0.000	1.205	0.893	33.3	6	-0.68	2	-1.16	0.888	0.0
1hlb	30	1	0.000	1.661	0.812	33.3	15	-0.29	2	-1.16	0.812	0.0
1ith-A	30	1	0.000	1.775	0.904	33.3	23	-0.10	2	-1.16	0.893	0.0
1mbs	30	18	1.823	-0.270	0.754	33.3	3	-0.99	2	-1.16	0.835	0.0
1myt	30	1	0.000	2.429	0.762	100.0	2	-1.16	2	-1.16	0.725	50.0
2lhb	30	1	0.000	1.976	0.563	33.3	12	-0.38	4	-0.86	0.460	0.0
4sdh-A	30	1	0.000	3.221	0.839	33.3	21	-0.14	2	-1.16	0.750	0.0
**Average**	**30.0**	**2.7**	**0.182**	**1.762**	**0.821**	**50.0**	**9.8**	**-0.62**	**2.2**	**-1.13**	**0.800**	**10.0**

ig_structal												

1bbd	61	1	0.000	2.304	0.605	16.7	27	-0.35	10	-0.78	0.554	0.0
1dfb	61	7	1.854	0.864	0.530	16.7	12	-0.70	4	-1.18	0.528	16.7
1fai	61	4	1.736	1.172	0.481	16.7	13	-0.66	4	-1.18	0.462	0.0
1fig	61	59	1.702	-2.283	0.349	0.0	21	-0.46	6	-1.00	0.504	0.0
1fpt	61	2	1.333	1.358	0.583	33.3	8	-0.88	4	-1.18	0.565	16.7
1fvd	61	1	0.000	2.112	0.606	16.7	22	-0.44	2	-1.48	0.574	0.0
1gig	61	1	0.000	2.787	0.547	16.7	10	-0.78	10	-0.78	0.469	0.0
1iai	61	1	0.000	2.103	0.644	33.3	8	-0.88	2	-1.48	0.613	16.7
1igf	61	2	1.774	1.353	0.607	16.7	31	-0.29	8	-0.88	0.591	0.0
1ikf	61	1	0.000	2.561	0.592	33.3	8	-0.88	3	-1.30	0.540	16.7
1jhl	61	1	0.000	1.380	0.333	16.7	41	-0.17	5	-1.08	0.286	0.0
1mcp	61	1	0.000	2.143	0.623	66.7	3	-1.30	2	-1.48	0.585	50.0
1mrd	61	1	0.000	2.305	0.379	16.7	51	-0.07	7	-0.93	0.264	0.0
1ngq	61	1	0.000	2.716	0.543	33.3	2	-1.48	2	-1.48	0.456	16.7
1opg	61	1	0.000	2.175	0.575	33.3	44	-0.14	3	-1.30	0.529	33.3
1tet	61	1	0.000	2.323	0.567	33.3	4	-1.18	4	-1.18	0.523	16.7
1vge	61	1	0.000	2.766	0.208	16.7	45	-0.13	26	-0.36	-0.020	0.0
2fb4	61	1	0.000	2.277	0.486	16.7	32	-0.27	13	-0.66	0.422	0.0
3hfl	61	1	0.000	2.648	0.243	33.3	29	-0.32	4	-1.18	0.055	16.7
7fab	61	1	0.000	2.941	0.614	50.0	6	-1.00	3	-1.30	0.531	33.3
**Average**	**61.0**	**4.5**	**0.420**	**1.900**	**0.506**	**25.8**	**20.9**	**-0.62**	**6.1**	**-1.11**	**0.452**	**11.7**

ig_structal_hires												

1fgv	20	1	0.000	2.310	0.724	50.0	6	-0.50	2	-0.98	0.633	0.0
1gaf	20	1	0.000	2.827	0.649	50.0	8	-0.38	2	-0.98	0.493	0.0
1kem	20	1	0.000	1.518	0.636	50.0	11	-0.24	3	-0.80	0.567	0.0
1nbv	20	10	1.719	0.169	0.399	0.0	7	-0.43	4	-0.68	0.452	0.0
1vge	20	1	0.000	2.334	0.385	50.0	15	-0.10	2	-0.98	-0.116	0.0
2fbj	20	1	0.000	2.532	0.725	50.0	5	-0.58	2	-0.98	0.614	0.0
8fab	20	1	0.000	2.487	0.295	50.0	17	-0.05	8	-0.38	-0.228	0.0
**Average**	**20.0**	**2.3**	**0.246**	**2.025**	**0.545**	**42.9**	**9.9**	**-0.33**	**3.3**	**-0.82**	**0.345**	**0.0**

lattice_ssfit												

1dkt-A	1995	1	0.000	7.349	-0.049	8.5	996	-0.30	242	-0.92	-0.087	8.0
1pgb	1997	1	0.000	13.649	0.138	17.1	1909	-0.02	60	-1.52	0.087	16.6
**Average**	**1996.0**	**1.0**	**0.000**	**10.499**	**0.045**	**12.8**	**1452.5**	**-0.16**	**151.0**	**-1.22**	**0.000**	**12.3**

lmds												

1b0n-B	498	1	0.000	2.819	0.066	20.4	336	-0.17	10	-1.70	0.038	18.4
1dtk	216	70	7.224	0.375	0.044	4.8	42	-0.71	24	-0.95	0.038	4.8
1shf-A	437	1	0.000	4.275	0.064	11.6	378	-0.06	2	-2.34	-0.004	9.3
4pti	344	3	9.434	2.570	0.098	23.5	220	-0.19	4	-1.93	0.063	20.6
**Average**	**373.8**	**18.8**	**4.165**	**2.510**	**0.068**	**15.1**	**244.0**	**-0.28**	**10.0**	**-1.73**	**0.034**	**13.3**

semfold												

1eh2	11442	61	12.125	2.342	0.070	13.6	6511	-0.25	434	-1.42	0.069	13.5
1pgb	11282	1	0.000	7.782	0.096	19.2	2	-3.75	2	-3.75	0.091	19.2
**Average**	**11362.0**	**31.0**	**6.063**	**5.062**	**0.083**	**16.4**	**3256.5**	**-2.00**	**218.0**	**-2.59**	**0.080**	**16.3**

moulder												

1c2r	301	1	0.000	2.803	0.774	73.3	10	-1.48	2	-2.18	0.768	70.0
1cid	301	1	0.000	2.759	0.753	53.3	38	-0.90	2	-2.18	0.748	53.3
1gky	300	1	0.000	4.713	0.828	90.0	11	-1.43	3	-2.00	0.819	89.7
1mup	301	1	0.000	1.993	0.847	73.3	11	-1.44	3	-2.00	0.845	73.3
2cmd	301	1	0.000	2.506	0.911	36.7	15	-1.30	6	-1.70	0.911	33.3
2pna	301	85	3.523	0.723	0.816	66.7	30	-1.00	4	-1.88	0.817	66.7
8i1b	301	1	0.000	2.106	0.842	46.7	16	-1.27	4	-1.88	0.840	46.7
**Average**	**300.9**	**13.0**	**0.503**	**2.515**	**0.824**	**62.9**	**18.7**	**-1.26**	**3.4**	**-1.97**	**0.821**	**61.9**

rosetta												

1a68	141	1	0.000	2.608	0.624	64.3	11	-1.11	3	-1.67	0.608	64.3
1aiu	141	15	1.385	0.805	0.777	7.1	40	-0.54	25	-0.75	0.776	7.1
1bk2	141	1	0.000	2.562	0.820	78.6	14	-1.00	3	-1.67	0.812	78.6
1bq9	141	2	9.242	1.895	0.544	50.0	131	-0.03	2	-1.85	0.532	50.0
1cc8	141	1	0.000	3.317	0.848	64.3	11	-1.11	2	-1.85	0.851	57.1
1ctf	141	1	0.000	4.288	0.783	28.6	30	-0.67	6	-1.37	0.780	28.6
1elw	141	22	3.619	1.082	-0.063	7.1	132	-0.03	12	-1.07	-0.070	7.1
1eyv	141	1	0.000	3.194	0.587	35.7	22	-0.80	2	-1.85	0.564	35.7
1gvp	141	1	0.000	3.018	0.540	21.4	61	-0.36	15	-0.97	0.514	21.4
1iib	141	1	0.000	6.093	0.595	71.4	9	-1.19	2	-1.85	0.629	71.4
1lou	141	1	0.000	2.664	0.741	71.4	8	-1.24	3	-1.67	0.731	64.3
1pgx	141	1	0.000	2.232	0.821	35.7	75	-0.27	5	-1.45	0.820	28.6
1rnb	141	8	13.461	1.445	0.440	21.4	89	-0.20	14	-1.00	0.427	21.4
1ten	141	1	0.000	4.986	0.866	92.9	5	-1.45	2	-1.85	0.876	85.7
1tul	141	5	0.842	2.002	0.763	64.3	16	-0.94	3	-1.67	0.755	57.1
1urn	141	1	0.000	2.452	0.694	64.3	9	-1.19	3	-1.67	0.680	57.1
1vie	141	1	0.000	3.470	0.791	71.4	2	-1.85	2	-1.85	0.781	71.4
256b	141	1	0.000	4.974	0.426	7.1	118	-0.07	42	-0.52	0.390	0.0
2ci2	141	80	10.219	0.075	-0.020	0.0	123	-0.06	71	-0.30	-0.022	0.0
**Average**	**141.0**	**7.6**	**2.040**	**2.798**	**0.609**	**45.1**	**47.7**	**-0.74**	**11.4**	**-1.41**	**0.602**	**42.5**

The performance scores for respective PDB IDs of the target proteins and their average are shown by individual generation methods. The number of decoy structures and single native structure (N), the rank of the native structure relative to decoy structures based on the calculated pseudo-energy (R_nat_), and the rest of the scores, described in the Methods, are shown.

**Figure 2:**
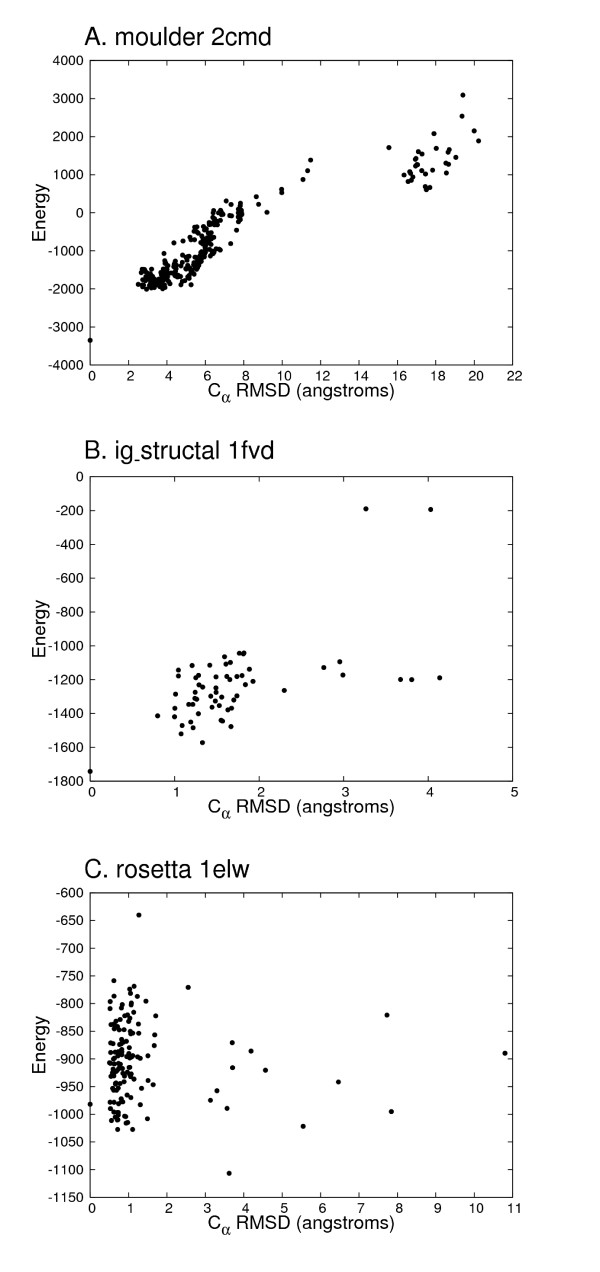
**Examples of the distribution of total pseudo-energy against C_*α *_RMSD**. Examples of the distribution of total pseudo-energy (Energy) against C_*α *_RMSD are shown according to the correlation coefficient (C.C.) value from the test result. The native structures are at 0.0 of C_*α *_RMSD. (A) 2cmd from the moulder decoy set (the best C.C. of 0.911). (B) 1fvd from the ig_structal decoy set (median C.C. of 0.606). (C) 1elw from the rosetta decoy set (the worst C.C. of -0.063).

As for the effectiveness for the individual decoy sets (Table 3), nonuniformity was observed, as mentioned in the Background. The best average Z-score was obtained for lattice_ssfit (10.499), and the worst for hg_structal (1.762). The average Z-score values were positive in all decoy sets. The best average C.C. and average F.E. were for moulder (0.824 of C.C. and 62.9% of F.E.), and the worst were for lattice_ssfit (0.045 of C.C. and 12.8% of F.E.). In Figure 2, three examples of energy distribution against C_*α *_RMSD are shown. The average C.C._decoy _of 4state_reduced (0.767), hg_structal (0.800) and moulder (0.821) were relatively high. The worst average C.C._decoy _(0.000) was obtained for lattice_ssfit. The average F.E._decoy _of 4state_reduced (61.5%) and moulder (61.9%) were significant, and the worst was for ig_structal_hires (0.0%).

### Comparison with other statistical potentials

We compared the performance of the DFMAC with 6 different state-of-the-art statistical potentials of DOPE [15], RAPDF [21], DFIRE [7], and PC2CA [16]. To exclude possible training biases, the target structures for comparison were restricted to the entries listed in our test set. Comparison of the rank of the native structures is shown in Table 4. DOPE, RAPDF, and DFIRE-A use residue-specific heavy atom representations. DFIRE-B uses the main-chain and C_*β *_atoms. PC2CA uses C_*α *_atoms and the side-chain center of mass. DFMAC uses main-chain atoms (N, C_*α*_, and C) per residue, while evaluation was carried out with generated pseudo atoms of C_*β*_, main-chain amino hydrogen (H) and carbonyl oxygen (O). In regard to the total number of correct identifications out of 11 total proteins, DOPE and DFIRE-A identified 10 native structures, followed by PC2CA (9 correct), DFMAC (8), RAPDF (7), and DFIRE-B (6). From this viewpoint, the performance of DFMAC was moderate. DOPE was also significant according to the averaged rank (4.0), followed by DFMAC (8.1), DFIRE-B (26.9) and DFIRE-A (40.0). RAPDF and PC2CA were similar (~60). Although the number and the types of targets applied here were limited and biased to a certain degree, DFMAC provided at least one better performance indexes against many other functions.

**Table 4:** Comparison of the function performances.

decoy set	protein	DFIRE-A	DFIRE-B	DOPE	RAPDF	PC2CA	DFMAC
4state_reduced	1ctf	1	1	1	1	1	1
4state_reduced	2cro	1	2	1	1	1	1
4state_reduced	4rxn	1	19	1	1	667	1
fisa	2cro	1	1	1	14	1	1
fisa_casp3	1bl0	1	3	1	1	1	8
lattice_ssfit	1dkt-A	1	1	1	1	1	1
lattice_ssfit	1pgb	1	1	1	1	1	1
lmds	1b0n-B	430	261	34	359	1	1
lmds	1dtk	1	5	1	116	2	70
lmds	1shf-A	1	1	1	1	1	1
lmds	4pti	1	1	1	157	1	3

average		40.0	26.9	4.0	59.4	61.6	8.1
correct		10	6	10	7	9	8

The rank of the native structure identified by respective functions is shown for the targets listed. The results of DFIRE-A, DFIRE-B and RAPDF were from the literature [7]. The results of DOPE were from [15]. The results of PC2CA were from [16]. The average rank (average) and the number of correctly identified native structures (correct) in 11 targets are shown.

Among the functions dealing with coarse-grained structure representation (DFIRE-B, PC2CA, and DFMAC), PC2CA had the largest number of correct identifications. We thus carried out additional comparison of DFMAC with PC2CA to identify the detailed relative performance of DFMAC. The performance of DFMAC on the targets of the test set, except for moulder and rosetta decoy sets, was compared with PC2CA results reported in the literature [16] (Table 5). Forty (78.4%) native structures were correctly identified by DFMAC from test targets, while PC2CA identified fewer native structures of 16 (31.4%). PC2CA and DFMAC had distinctive performances for the respective decoy sets. For example, PC2CA showed better performances with all of the averaged indexes (correct, C_*α *_RMSD, Z-score, C.C., and F.E.) for the lmds decoy set, while DFMAC was better for 4state_reduced, ig_structal and ig_structal_hires. All of the summarized indexes were better with DFMAC. Although the number and kinds of decoy sets used here were limited in number and compilation of a variety of characteristics, the performance of DFMAC could be at least roughly similar to one of the state-of-the-art functions, PC2CA.

**Table 5:** Comparison of PC2CA and DFMAC functions on the test set.

		PC2CA					DFMAC				
			
decoy set	total	correct	C_*α *_RMSD	Z-score	C.C.	F.E.(%)	correct	C_*α *_RMSD	Z-score	C.C.	F.E.(%)
4state_reduced	3	2	0.7	1.4	0.59	53.4	3	0.0	3.5	0.77	62.6
fisa	1	1	0.0	7.3	0.17	22.0	1	0.0	4.2	0.28	24.0
fisa_casp3	2	2	0.0	4.4	-0.02	10.4	1	3.7	3.0	0.22	19.6
hg_structal	10	5	0.8	1.3	0.70	53.3	9	0.2	1.8	0.82	50.0
ig_structal	20	0	2.2	-0.8	0.31	18.3	15	0.4	1.9	0.51	25.8
ig_structual_hires	7	0	2.6	-0.2	0.32	0.0	6	0.2	2.0	0.54	42.9
lattice_ssfit	2	2	0.0	3.9	0.02	11.1	2	0.0	10.5	0.04	12.8
lmds	4	3	1.6	3.7	0.10	19.5	2	4.2	2.5	0.07	15.1
semfold	2	1	0.2	2.7	0.05	13.0	1	6.1	5.1	0.08	16.4

Summary	51	16	1.5	0.9	0.35	24.1	40	0.9	2.6	0.50	33.1

Only the results for targets listed in our test set are compiled and shown. The number of correctly identified native structures (correct) out of the total targets (total) is shown by individual generation methods. The averages of C_*α *_RMSD, Z-score, C.C., and F.E. for the respective decoy sets are also shown. In the "Summary" column, the sum of total and correct counts, and the averages of C_*α *_RMSD, Z-score, C.C., and F.E. over the respective protein targets, are shown. The results of PC2CA [16] were used and the respective score averages were calculated.

### Contributions of the function components to performance

DFMAC consists of six pseudo-energy calculating components. We evaluated the contribution of each component to the structure discrimination ability. The original DFMAC function was compared to functions without any of the components on the test set (Table 6). A significant increase in average C_*α *_RMSD without the DABG component, followed by the SURR component, was observed, indicating the major contributions of the two components. The deficiency of discrimination ability without these two components was similarly observed for most of the other indexes, supporting the significance of these components. The influence of any one of 4 other components was smaller, and most indexes remained similar to the original function; however, the averaged rank of the native structures increased without OMDA, indicating a certain degree of contribution. When cross validation with tuned parameters was carried out on the training set without any one of the six components, no improvement in average C_*α *_RMSD was observed (data not shown). This result also suggests the requirement of all six components. Additionally, the inclusion of HBND, PPDA, and OMDA components is expected to have discriminative ability for a possible chain modeling application.

**Table 6:** Effects of the omission of each energy calculation component from the DFMAC function.

omitted component	R_nat_	C_*α *_RMSD	Z-score	C.C.	F.E.(%)	logP_B1_	logP_B10_	C.C._decoy_	F.E._decoy_(%)
none	6.8	1.174	2.630	0.559	38.7	-0.75	-1.41	0.518	25.1
DIST	6.9	1.087	2.687	0.547	38.1	-0.76	-1.40	0.508	25.4
DABG	16.2	2.444	2.062	0.554	36.9	-0.69	-1.40	0.507	25.8
HBND	6.0	1.301	2.523	0.558	39.3	-0.76	-1.44	0.520	26.5
PPDA	6.7	1.197	2.617	0.558	39.1	-0.75	-1.44	0.518	25.0
OMDA	12.0	1.036	2.582	0.555	39.5	-0.76	-1.42	0.517	25.4
SURR	11.6	1.519	2.737	0.487	35.2	-0.68	-1.37	0.442	22.0

The measures are as described in Table 3. The average scores over the test set are shown by the omitted component.

## Discussion

A knowledge-based decoy discriminatory function (DFMAC) was successfully developed. The DFMAC function requires the input data of the coordinates of only three main-chain atom types (N, C_*α*_, and C) per each amino-acid residue. The function is formalized as the combination of six pseudo-energy calculating components. Each component evaluates a different feature of a protein. The native or near-native structures in various types of decoy sets were recognized with high accuracy. The discrimination ability was nearly comparable to other state-of-the-art coarse-grained or all-atom-type scoring functions.

One notable feature of the function is the simplicity of the required representation of the model structures, consisting of only three main-chain atom coordinates per residue. Such input structural data is beneficial for structure modeling. Because the side chain conformation need not be scanned, the main chain conformation scan could be facilitated. The scanning of different folds for evaluation of sequence-fold compatibility could also be facilitated. The construction of an all-atom model is possible by assigning side-chain coordinates to a reasonable main-chain model.

The considerable accuracy of the whole function was derived mainly from the DABG component. This component evaluates the relative orientation of the pseudo C_*β *_atom against the associated C_*α *_atom between two residues. The recognition of acceptable orientation conversely restricts the degree of freedom of main-chain conformation and side-chain orientation. Thus, more accurate fold recognition could be provided than a simple distant dependent function among, for example, C_*α *_atoms. The all-atom distant-dependent-type functions would implement similar or more accurate fold recognition, by judging acceptable main-chain/side-chain orientation with multiple distances per residue. Although our representation of the structure is far simpler, an alternative structure recognition mechanism would be implemented, at least partially. The effectiveness of orientation-dependent potentials was also shown by Buchete et al. [9,10]; the interaction centers were defined for respective side chains and peptide bonds, and six parameters were used to express a single pairwise interaction. In our case, evaluation was performed with more limited conditions, using only a single point per residue and as few as 4 parameters per pairwise interaction between the points; however, the DABG component of DFMAC was able to provide considerable discrimination capacity.

Compared with the DABG and SURR function components, the contributions of 4 other components were smaller; however, the DABG component does not evaluate local main-chain conformation within a 2-residue distance. The SURR component also does not evaluate local main-chain conformation. Thus, HBND, PPDA, and OMDA components were implemented to recognize the allowed conformation for possible model building experiments, although little pullback of discrimination was observed. Additionally, since many decoy structures already had reasonable local conformations, significant contribution of these components might not be observed. Improvement of these components to more harmless and versatile ones could help refine the overall function.

Nine of the 77 test targets failed in native or near-native structure identification. The IDs were 1bl0, 1dtk, 4pti, 1eh2, 2pna, 1bq9, 1elw, 1rnb, and 2ci2. Many plausible reasons for the failure of (near-) native structure recognition can be found [15,16]. The distorted geometries of 1dtk could harm the scoring of the native structure [16]. The presence of other chains in the crystal structure is also a possible reason for the failure [16]. The difficulty of 1bl0, which was bound to DNA, might also have failed because of the complex structure. The 1rnb is also a complex of protein and small molecules. Interaction with the metal ion might also make discrimination harder. The crystal structure of 1bq9 contains Fe(III) ion. NMR structures are often suggested to be difficult to identify [16]. The 1dtk, 1eh2, and 2pna are NMR structures, and a difficulty might arise for that reason. The difficulty with smaller proteins is also frequently discussed. We tested the correlation of protein size and accuracy, and we also found a difficult tendency for smaller polypeptides (data not shown). The failure of 1dtk (57 residues), 4pti (58 residues), 1bq9 (51 residues), and 2ci2 (62 residues) might have resulted. The possible origin of failure of the remaining 1elw top structure was not apparent.

The capacity of DFMAC to recognize a correct fold among different folds, which are separated in the structure space, is not apparent. In the rosetta decoy set [12], each target consists of 20 refined native structures and the 100 lowest scoring models out of ~10,000 *de novo *predicted models among a variety of conformations. Evaluation of the test set with DFMAC resulted in correct identification of 68.4% (13/19) native and 78.9% (15/19) near-native structures; therefore, the capacity for fold recognition which could support *de novo *structure prediction might be expected.

The high C_*α *_RMSD of the top structures was frequently observed for some decoy sets, such as lmds or rosetta. One of our next challenges is to improve our function to cover these "difficult" decoy sets. The introduction of a high-resolution structure dataset for database construction [17] and the development of an additional all-atom-type evaluation system are possible solutions. Additionally, since the function was mainly implemented with pairwise interactions, a frustrated structure, which consists of locally allowed pairwise interactions, might be positively evaluated. Based on these considerations, further improvement of the function in decoy or fold discrimination ability is now in progress.

## Conclusion

A novel knowledge-based decoy discrimination function, DFMAC, was successfully constructed. Despite the simple representation of protein structure models of input data, the discrimination ability was nearly comparable to other coarse-grained and all-atom-type functions. The orientation-dependent pseudo-energy calculating component (DABG), in addition to the component for the number of surrounding atoms (SURR), was found to be significantly effective for performance of the function. A variety of applications of the function to support activities such as structure prediction is expected.

## Methods

### Overview of the function formulation

The function for total energy calculation is formulated as the sum of six weighted pseudo-energy terms:

$$\begin{array}{c} E_{total}=W_{DIST}*E_{DIST}+W_{DABG}*E_{DABG}+W_{HBND}*E_{HBND}\\ +W_{PPDA}*E_{PPDA}+W_{OMDA}*E_{OMDA}+W_{SURR}*E_{SURR} \end{array}$$

where *E*_*total *_is the total pseudo-energy, the "*w*" and "*E*" with subscripts on the right side of the equation are the weights and pseudo-energy calculation components, respectively. The subscripts of the terms on the right side correspond to the six respective types of pseudo-energy components, dealing with the distances between two C_*α *_atoms (referred to as DIST), the relative orientation of the vector of C_*α *_to pseudo-C_*β *_atom coordinates between two residues (DABG) (Figure 1A), hydrogen bonds between the main-chain amino hydrogen and the main-chain carbonyl oxygen atoms (HBND) (Figure 1B), the *ψ*-*φ *dihedral angles (PPDA), the *ω *dihedral angle (OMDA), and the number of the surrounding C_*α *_atoms (SURR). Each pseudo-energy component was calculated referring to a specifically precompiled database derived from the known protein structures. The formulation details are described below.

### Preparation of database from native protein structures

Databases for pseudo-energy calculation components, which evaluate individual structural features, were derived from 3,313 nonhomologous (less than 25% homology) protein structures with a resolution of better than 2.0 Å and *R*-factors of better than 0.25. The list of the proteins (compiled on June 23, 2007) was provided by PISCES server [22]. Structural data with atomic coordinates were from the protein data bank (PDB) [23]. The coordinates of the three main-chain atom types of amino nitrogen (N), alpha carbon (C_*α*_), and carbonyl carbon (C) of all 3,313 structures were used for database construction. The database was compiled for each of the pseudo-energy calculating components. All databases, except for SURR, were built considering the combination of the subject and object amino acid types. Thus, 400 sub-databases were generated for each component. The SURR database consists of 20 sub-databases for respective amino acid types of subject residues. The domains of parameter(s) were divided into uniform-sized bins. All measurements, which met the specific criteria below, were classified and counted in corresponding bins. For the DIST database, C_*α *_pairwise distances were compiled with 64 bins in the range from 0.0 Å to 21.0 Å. Residue pairs within a certain distance in the amino acid sequence were not included. This pair inclusion range in the sequence was tuned by the procedures described below. The DABG database was derived using C_*α *_atom coordinates and pseudo-C_*β *_atom coordinates, which were generated based on the coordinates of N, C_*α*_, and C atoms of the respective residues. Four parameters of the distance between the two C_*α *_atoms, and the *α*, *β*, and *γ *angles were applied to represent unique relative orientation between two C_*α*_-pseudo-C_*β *_atom vectors of the residues (Figure 1A). The criteria for residue inclusion and distance parameter range were the same as DIST. The pair inclusion range in the sequence were also tuned as described below. The *α *and *β *angles (0° to 180°) were divided into 16 bins, and the *γ *angle (-180° to 180°) was divided into 32 bins. Data were compiled into four-dimensional sub-databases. The HBND database was derived using the coordinates of pseudo amino hydrogen atoms (H) and pseudo carbonyl oxygen atoms (O), which were generated using N, C_*α*_, and C main-chain atom coordinates. (Note: The N-terminal pseudo-H and C-terminal pseudo-O of each fragment could not be generated and compiled because of the absence of their preceding and following residues, respectively.) The three parameters of the distance between pseudo-H and pseudo-O atoms, and the *η *and *θ *angles were calculated (Figure 1B). The distance, ranging from 1.7 Å to 2.9 Å, was divided into 4 bins. Either of the *η *and *θ *angles, ranging from 0° to 180°, was divided into 16 bins. Data were compiled into three-dimensional sub-databases. The PPDA database was derived using the *ψ *and *φ *main-chain dihedral angles of the peptide bond. We constructed a database dealing with the *ψ *angle of one residue and the *φ *angle of the next residue, which is different from the standard Ramachandran-type representation (i.e. *φ *and *ψ *angles for a single residue). Thus, 400 variations of sub-databases were generated for respective permutations of amino acid types of two adjacent residues. Either of the angles, ranging from -180° to 180°, was divided in 64 bins. Data were compiled into two-dimensional sub-databases. The OMDA database was derived using the *ω *dihedral angles of peptide bonds. Thus, the variation of sub-databases was 400, corresponding to the respective amino acid permutations. The *ω *angle, ranging from -180° to 180°, was divided in 128 bins. The SURR database is related to the degree of embedding of the residue in a molecule. The number of C_*α *_atoms in the sphere with a certain radius from the central C_*α *_atom coordinate of the focusing residue was counted as the surroundings. The radius was also tuned as described below. The count was classified into the respective bins of corresponding counts. Data were compiled into 20 sub-databases, associated with the respective amino acid types.

### Pseudo potential energy calculation

Each of the above six pseudo-energy calculation components was derived based on the Boltzmann law [5]. The pseudo-energy (*E*_*s*_) for a state "*s*" was calculated with the following equation:

$$E(s)=-\ln(\frac{N_{obs}(s)}{N_{\exp}(s)})$$

where *N*_*obs*_(*s*) and *N*_*exp*_(*s*) represent the number of observed and expected counts for the state (*s*), respectively. Since the energy values were utilized as relative scores throughout the analyses, the factor of *kT *was not included in the formula with the assumption of constant temperature. Unless otherwise stated, counts in the corresponding bin of the database were used as *N*_*obs*_(*s*). The expected count is also referred to as the reference, which was from the distribution without the interaction focused on for the component. The total pseudo-energy for each component (*E*_*COMP*_) was calculated as:

$$E_{COMP}=\sum_{s}^{all}E(s)$$

The criteria for inclusion in the energy summation were defined by individual components. The parameter set, bin size, and bin distribution were the same as the database construction conditions described above. The specific energy calculation conditions for each component were as described below. In the case of DIST component calculation, the measured distances between two C_*α *_atoms of the respective residue pairs were used as specific states. In consideration of the finite size of proteins, the corresponding *N*_*exp*_(*s*) was calculated as [16]:

*N*_exp_(*d*) = a*d^2 ^*exp(-(*d*/*b*)^*c*^)* Δ*d*

where *d *is the central value of the corresponding distance bin, Δ*d *is the bin size, and *a*, *b*, and *c *are constants. Constant *a *was adjusted as follows: the sum of the counts (*A*_*sum*_) in the database was calculated for bins ranging from 56^th ^to 64^th ^(i.e. the distance ranging from 18.05 Å to 21 Å). On the other hand, the integral of *N*_*exp*_(*d*) (*A*_*integ*_) for the same distance range was calculated assuming that *a *is 1. The value of (*A*_*sum*_/*A*_*integ*_) was then re-assigned to the *a *constant. The *b *and *c *constants were scanned and tuned with the procedure described below. The pairs, which have distances ranging from 0 Å to 15.75 Å, and a certain degree of sequence separation of |*i*-*j*| between *i*^th ^and *j*^th ^residues in the amino acid sequence, were subjected to energy calculation. The minimum limit of sequence separation was subjected to tuning. When the count of the bin in the database was 0, a penalty energy value was alternatively assigned. This was also tuned. For DABG energy calculation, the parameters of the distance, and *α*, *β*, and *γ *angles were calculated with pseudo-C_*β*_, as described above in the database preparation (Figure 1A). *N*_*obs *_was the average count of the bins in the database with the center at the position corresponding to the measured parameters of the distance, and *α*, *β*, and *γ *angles. Averaging with bins extending to certain position ranges for both sides was applied for each parameter. These ranges were also tuned. *N*_*exp *_was calculated as follows: assuming that the C_*α*_-pseudo-C_*β *_vector orientation is random, the probability density function (*P*) for *α *or *β *parameters is:

$$P(\alpha \text{ or }\beta )=\sin(\frac{\left(\alpha \text{ or }\beta \right)}{180}*\pi )/2$$

The *P*(*γ*) is supposed to distribute uniformly along the *γ *angle; thus, *N*_*exp*_(*d*, *α*, *β*, *γ*) is formulated as:

$$\begin{array}{l} N_{\exp}(d,\alpha ,\beta ,\gamma )=\\ N_{obs}(d)*\frac{\sin(\frac{\alpha }{180}*\pi )}{2}*\frac{\sin(\frac{\beta }{180}*\pi )}{2}*\frac{\Delta \gamma }{360} \end{array}$$

where *α *and *β *are the center of the corresponding bin, Δ*γ *is the bin size of *γ*. *N*_*obs*_(*d*) of the DIST component was used as a substitute for *N*_*exp*_(*d*). Residues with the same sequence separation as DIST were evaluated. The penalty value for the 0 of the average count was subjected to tuning. The method of HBND energy calculation was the simpler version employed by Kortemme et al. [8]. The function evaluated only the main-chain hydrogen bond between pseudo-H and pseudo-O atoms using three parameters (Figure 1B). As described for the DABG component, the probability density function under the random orientation of N-H or C-O vectors is represented as:

$$P(\eta \text{ or }\theta )=\sin(\frac{\left(\eta \text{ or }\theta \right)}{180}*\pi )/2$$

The expected distance distribution probability *P*_*exp*_(*d*) is assumed to have a similar form to the finite ideal gas reference state [7] as:

*P*(*d*) = c*d^1.6^

where *d *is the center of each distance bin. Constant *c *was adjusted to make the sum of the probability for 4 total bins of possible conditions to 1. *N*_*exp*_(*d*, *η θ*) for HBND component was thus expressed as:

$$\begin{array}{l} N_{\exp}(d,\eta ,\theta )=\\ N_{total}*c*d^{1.6}*\frac{\sin(\frac{\eta }{180}*\pi )}{2}*\frac{\sin(\frac{\theta }{180}*\pi )}{2} \end{array}$$

where *N*_*total *_is the total observed count compiled in the corresponding pairwise sub-database. The penalty value was tuned. In the case of PPDA energy calculation, equal distribution on the *ψ*-*φ *plane was assumed for the expected probability. Thus, *N*_*exp*_(*ψ*,*φ*) for PPDA component is:

*N*_exp _(*ψ*, *ϕ*) = *N*_total_* Δ*ψ*/360* Δ*ϕ*/360

where *N*_*total *_was the total observed count compiled in the corresponding pairwise sub-database, and Δ*ψ *and Δ*φ *were the bin sizes of the respective angles. The penalty value was tuned. OMDA energy was calculated similarly with equal distribution along the *ω *axis assumed for the expected probability. Thus, *N*_*exp*_(*ω*) was:

*N*_exp_(*ω*) = *N*_total_* Δ*ω*/360

where *N*_*total *_was the total observed count, and Δ*ω *was the bin size of the *ω *angle. The penalty value was tuned. The concept of SURR energy was similar to the solvation potential by Jones [6]. The distribution of the observed count of surrounding C_*α *_atoms was compiled in a procedure similar to the database construction for each amino acid type. The resultant database was standardized by each of the sub-databases, and then used as the expected count of *N*_*exp*_(*n*) as:

$$N_{\exp}(n)=N_{aa}*\frac{N(n)}{N_{tot}}$$

where *n *is the number of surrounding C_*α *_atoms in a sphere, *N*_*aa *_is the sum of the counts for a specific amino acid type over the surrounding numbers, *N*_*tot *_is the sum of the counts of all residues of all structures over the surrounding numbers, and *N*(*n*) is the count of the specific number of surroundings (*n*) for all residues of all structures. The radius of the sphere and the penalty value were tuned.

### Decoy sets

The Decoys 'R' Us decoy database , which was compiled by Samudrala and Levitt [18], was used for parameter tuning and function evaluation. For the l30 target in the fisa_casp3 decoy set, 1ck2 from PDB was used as the native structure [16]. C_*α *_RMSD of the l30 native structure in the original list (1.882 Å) was used as the native C_*α *_RMSD. The moulder decoy set [15,20] and the all atom decoy set from Rosetta@home  (or "rosetta"), which were from the homepages of the Sali lab. and Baker lab., respectively, were also used. The rosetta set contains well-scoring Rosetta protein models and their native crystal structures for 59 proteins, without 3 NMR structures. We repacked 141 structures of one native PDB structure, the 20 refined native structures, the 100 lowest scoring models out of ~10,000 total models, and 20 random models, per protein into a single target entry for use.

### Determination of initial parameter values

The 7 decoy sets of 4state_reduced, fisa, fisa_casp3, hg_structal, ig_structal, ig_structal_hires, and lmds from the Decoys 'R' Us database [18] were used to search the initial parameter values. Several parameters were scanned at a time, and the best values of the parameters were determined successively. The procedure was repeated until all the parameters were scanned. Following are *the function components *and their associated parameters determined by the above procedure: *the DIST component*, the distance range for database construction and scoring, the sequence separation for database construction and scoring, the upper distance limit for scoring, the function form of *N*_*exp*_, the values of *b *and *c*, the lower distance limit for determination of the *a *value, and the penalty value; *the DABG component*, the distance range for database construction and scoring, the sequence separation for database construction and scoring, the upper distance limit for scoring, the range of neighboring bins for averaging the counts, and the penalty value; *the HBND component*, the distance range for database construction and scoring, and the penalty value; *the PPDA and OMDA components*, the penalty values; *the SURR component*, the radius of the sphere for database construction and scoring. Bin sizes for all of the components were determined appropriately. Each time a new parameter value was applied, the weight parameters *w *for the respective energy components were scanned, and the performance was evaluated with the temporary optimized weight values. Because the total pseudo-energy was used as a relative index value (not as an absolute energy), *w*_*DIST *_was fixed as 1 and the remaining 5 weights were scanned. The searching procedure for the weights was as follows: firstly, all of the combinations of discrete weight values, evenly spaced in a logarithmic scale (0.01 to 31.6, 15 steps), were evaluated, and the weight set with the best discrimination performance was selected. Then, another more precise cycle was carried out around the set of weight values determined by the previous scan (0.56- to 1.78-fold the previous weight value, 11 steps). The optimized weights for the function were 1, 0.316, 0.141, 0.200, 0.00562, and 0.178 for the components of DIST, DABG, HBND, PPDA, OMDA, and SURR, respectively.

### Function tuning by cross validation

The temporary optimized function by the previous procedure was further tuned through the cross-validation procedure. The 231 targets from the Decoys 'R' Us database, and the decoy sets of moulder and rosetta, were split into 154 (2/3 of total) for the training set and 77 (1/3 of total) for the test set according to their temporarily-assigned serial numbers. The targets were listed in the sequence of the decoy sets, and serial numbers of multiples of 3 were selected as the test set, and the rest of the targets were the training set. Thus, each decoy set was included in both training and test sets with a roughly equal ratio, without any intentional bias. The parameters were tuned by 10-fold cross validation. The above training set was divided into 10 segments, with new identification numbers in cyclic order. The function with temporally optimized weights was evaluated on one remaining target segment. The average C_*α *_RMSD of top structures from 10 evaluations of all combinations of training and evaluation was set as the performance index. The weights of the function components, with a set of updated parameter values, were optimized for 9 target segments to the minimum C_*α *_RMSD average. Weights were optimized by the following iterative cycles of scanning: the combination of discrete values, equally spaced in the logarithmic scale for each of the six weights, was scanned in a cycle. The 1^st ^cycle was 5 steps of 0.01 to 100. The following 4 cycles were repeated for the 8 best weight sets found by the 1^st ^cycle, which were separated by at least a 6-step distance. Each of the 2^nd ^to 5^th ^cycles evaluated the 3 steps of the parameters, i.e. *w*^-*r*^, *w*, and *w*^*r*^, where *w *was the weight value selected by the previous cycle, and *r *was the factor of the scanning range. If any one of the selected parameter values was not the previous one (i.e. the optimum was not at the center of scanning), the same cycle was repeated again with the selected parameter values. The *r *values of 2^nd^, 3^rd^, 4^th^, and 5^th ^cycles were 3.16, 1.78, 1.33, and 1.15, respectively. The final weight values tuned by the procedure were 1.00, 0.662, 0.765, 0.0372, 1.02, and 43.0 for the components of DIST, DABG, HBND, PPDA, OMDA, and SURR, respectively. The tuned parameters, the order of the parameters successively scanned, and the initial, scanned, and final values are listed in Table 1.

### Performance measures

The performance measures and their definitions are as follows: *C_*α *_RMSD*, the root mean square deviation of the C_*α*_-C_*α *_pairs between the native structure and the model with the best energy; *Z-score*, the score of the native structure, which was calculated under the standard definition [11] (Note: the positive value corresponds to the lower (better) energy than average.); *C.C*., Pearson's correlation coefficient among the structures including the native and the decoys; *F.E*., the fraction of the top 10% lowest C_*α *_RMSD structures in the top 10% best-energy structures among the structures, including the native and the decoys; *R*_*B1*_, the C_*α *_RMSD rank of the best-energy structure among the decoy structures; *logP*_*B1*_, the common logarithm of the probability of selecting the best decoy structure, where *P*_*B1 *_= *R*_*B1*_/(number of decoy structures); *R*_*B10*_, the lowest C_*α *_RMSD rank in the 10 best-energy decoy structures among the decoys; *logP*_*B10*_, the common logarithm of the probability of selecting the best decoy structure in the 10 best-energy decoy structures, where *P*_*B10 *_= *R*_*B10*_/(number of decoy structures); *C.C*._*decoy*_, the C.C. among the decoys; *F.E*._*decoy*_, the F.E. among the decoys.

## Authors' contributions

YM conceived the project, designed the function, carried out the computational experiments, and drafted the manuscript. NI provided intellectual guidance and mentorship. Both authors read and approved the final manuscript.
